# Human Skeletal Muscle Mitochondria Responses to Weight Loss Induced by Bariatric Surgery or Lifestyle Intervention

**DOI:** 10.1111/apha.70150

**Published:** 2026-01-08

**Authors:** Birgitta W. van der Kolk, Sini Heinonen, James W. White, Anita Wagner, Jari E. Karppinen, Sina Saari, Maheswary Muniandy, Simo Metsikkö, Eugène T. Dillon, Per‐Henrik Groop, Tuure Saarinen, Carel W. Le Roux, Kirsi A. Virtanen, Neil G. Docherty, Eija Pirinen, Anne Juuti, Kirsi H. Pietiläinen

**Affiliations:** ^1^ Research Program for Clinical and Molecular Metabolism, Faculty of Medicine, University of Helsinki Helsinki Finland; ^2^ Department of Internal Medicine and Rehabilitation Helsinki University Hospital Helsinki Finland; ^3^ Diabetes Complications Research Centre, School of Medicine, Conway Institute of Biomolecular and Biomedical Research, University College Dublin Dublin Ireland; ^4^ Department of Biomedical Sciences College of Medicine and Health, University of Birmingham Birmingham UK; ^5^ Chair for Molecular Nutritional Medicine, Technical University of Munich TUM School of Life Sciences Weihenstephan Freising Germany; ^6^ Mass Spectrometry Resource, Conway Institute of Biomolecular and Biomedical Science, University College Dublin Dublin Ireland; ^7^ Folkhälsan Research Center, Biomedicum Helsinki Helsinki Finland; ^8^ Department of Nephrology University of Helsinki and Helsinki University Hospital Helsinki Finland; ^9^ Department of Diabetes Central Clinical School, Monash University Melbourne Victoria Australia; ^10^ Baker Heart & Diabetes Institute Melbourne Victoria Australia; ^11^ Department of Gastrointestinal Surgery Abdominal Center, HUS and University of Helsinki Helsinki Finland; ^12^ Turku PET Centre, Turku University Hospital Turku Finland; ^13^ Turku PET Centre, University of Turku Turku Finland; ^14^ Faculty of Medicine, Research Unit of Biomedicine and Internal Medicine, University of Oulu Oulu Finland; ^15^ Medical Research Center Oulu, Oulu University Hospital and University of Oulu Oulu Finland; ^16^ Biocenter Oulu, University of Oulu Oulu Finland; ^17^ Healthy Weight Hub, Abdominal Center, Helsinki University Hospital Helsinki Finland

**Keywords:** bariatric surgery, lifestyle intervention, mitochondria, skeletal muscle, type 2 diabetes, weight loss

## Abstract

**Aim:**

We investigated how weight loss induced by bariatric surgery or lifestyle intervention affects skeletal muscle mitochondrial metabolism.

**Methods:**

We studied two weight‐loss cohorts: RYSA (BMI ≥ 35 kg/m^2^; *n* = 39, including 18 with diabetes) undergoing bariatric surgery, and CRYO (BMI ≥ 30 kg/m^2^; *n* = 19) undergoing a lifestyle intervention with a low‐calorie diet. Assessments were performed at 5–6 and 12 months and included muscle proteome (LC–MS/MS), mitochondrial biogenesis by mtDNA amount (qPCR), number and morphology (transmission electron microscopy) in both cohorts, and mitochondrial oxidative capacity (high‐resolution respirometry) in the surgery cohort.

**Results:**

Both cohorts achieved clinically meaningful weight loss, greater following surgery (24.4% vs 9.0% at 12 months). Per 1% weight loss, bariatric surgery was associated with significant downregulation of glycolysis pathways at 12 months. OXPHOS complex subunit proteins were associated with upregulation in individuals without diabetes but downregulation in those with diabetes. Lifestyle intervention was associated with downregulated OXPHOS complex subunits at 5 months. Mitochondrial morphology remained unchanged, while mtDNA amount correlated negatively with weight loss percentage in both cohorts. In the surgery cohort, complex I and complex I + II‐mediated respiration increased 3.2‐ and 2.9‐fold at 12 months, reflecting improved oxidative capacity.

**Conclusion:**

Bariatric surgery was associated with increased skeletal muscle mitochondrial respiration despite unchanged morphology and reduced mtDNA amount, whereas lifestyle‐induced weight loss showed a transient downregulation of OXPHOS‐related proteins with other mitochondrial markers remaining stable. Surgery‐induced weight loss may reflect improved mitochondrial efficiency in skeletal muscle, potentially influenced by diabetes status. Long‐term functional mitochondrial adaptations after weight loss require future studies.

**Trial Registration:**

RYSA: ClinicalTrials.gov ID NCT02882685; CRYO: ClinicalTrials.gov ID NCT01312090

## 1 Introduction

With over 2.5 billion people affected in 2022 [1], overweight or obesity have become a major global public health challenge, contributing to a wide range of complications, including type 2 diabetes (T2DM), cardiovascular diseases, and some cancers [2].

Reducing body weight substantially lowers health risks for individuals with obesity. Even a moderate weight loss of 5%–10% through caloric restriction enhances the metabolic profile [3]. Despite these benefits, sustaining weight loss through lifestyle modifications proves challenging, with relatively low success rates [4]. In contrast, bariatric surgery, though more invasive, yields more substantial and sustainable weight loss and frequently results in improvement or remission of type 2 diabetes and other associated complications [5].

Obesity and weight loss both affect cellular metabolism. Mitochondria, essential for cellular energy conversion, undergo many changes in obesity in skeletal muscle [6], including lower respiratory capacity, downregulated mitochondria‐related transcription [7], reduced mitochondrial amount and mitochondria‐related protein levels, impaired mitochondrial dynamics, and increased ROS production [8]. While weight loss improves metabolic health, it also reduces muscle mass and resting energy expenditure [9], raising questions about its effects on mitochondrial function. Notably, the impact of different weight loss methods, such as bariatric surgery versus lifestyle interventions including low‐calorie diets, on mitochondrial function remains underexplored.

Bariatric surgery may enhance skeletal muscle mitochondrial function post‐operation, particularly long‐term (for extensive review, see [10]). Following Roux‐en‐Y Gastric Bypass (RYGB), mitochondrial respiration increased without changes in mitochondrial quantity or oxidative phosphorylation (OXPHOS) complex subunit protein levels and mitochondrial respiration further increased with an exercise program post‐surgery [11]. Improvements in mitochondrial network and morphology, reduced fission, and constant mitochondrial amount have also been observed [12]. RYGB has also increased ADP‐stimulated coupled mitochondrial respiration and phosphate/oxygen (*P*/*O*) ratio when normalized to citrate synthase in isolated mitochondria, indicating improved mitochondrial efficiency [13]. A recent meta‐analysis linked surgery to increased mitochondrial amount and Complex I‐linked respiration [14]. However, some studies report unchanged respiration [15, 16], mitochondrial amount [16], or gene expression of biogenesis markers [17] following surgery. Downregulated TCA cycle and OXPHOS gene expression were found without altered protein expression [18]. Overall, muscle mitochondrial metabolism appears to benefit from surgery, but results vary by follow‐up duration, metabolic status including diabetes status, and exercise levels [10].

Following lifestyle or diet‐induced weight loss, most studies show limited or no changes in skeletal muscle mitochondrial metabolism. The CALERIE study, which applied 25% caloric restriction for 6 months to 2 years [19, 20, 21], found increased mitochondrial biogenesis at 6 months in overweight individuals [19]. After 12 months, mtDNA amount decreased, mitochondrial biogenesis remained unchanged and there were no changes in in vivo mitochondrial O_2_ uptake or ATP fluxes [21]. Overall, the prevailing trend is that lifestyle‐induced weight loss does not consistently reverse the decreased mitochondrial oxidative capacity observed in obesity [10].

However, comparisons between lifestyle and surgery studies are difficult to interpret because follow‐up durations differ and the mitochondrial markers assessed vary across studies. Here, we retrospectively examined changes in mitochondria‐related profiles in skeletal muscle following both bariatric surgery and lifestyle weight‐loss interventions, up to 12 months post‐intervention. To support comparisons between the two cohorts, we performed a uniform set of high‐quality analyses on the muscle proteome and mitochondrial markers across both cohorts. In addition, we measured mitochondrial respiration in the surgery cohort and accounted for baseline type 2 diabetes. This approach helped minimize technical variability and enabled clearer interpretation of longitudinal changes within each cohort.

## 2 Materials and Methods

### 2.1 Study Participants

#### 2.1.1 RYSA Study

The ongoing clinical study RYSA is a parallel‐group randomized controlled trial comparing RYGB to One‐Anastomosis Gastric Bypass (OAGB) [22, 23].

A total of 121 adults (BMI ≥ 35 kg/m^2^) were randomly assigned (1:1 ratio) at Helsinki and Oulu University Hospitals in Finland to undergo either RYGB or OAGB. Baseline measurements were conducted 8 weeks pre‐surgery, followed by a 4‐ to 6‐week low‐calorie diet (800–1000 kcal/day). Surgical procedures maintained consistent bypassed intestine lengths. Follow‐up measurements were done at 6 and 12 months post‐surgery. We analyzed data from a maximum of 39 participants (RYGB: *n* = 19; OAGB: *n* = 20), including 18 individuals with type 2 diabetes, for whom we had skeletal muscle mtDNA amount data from at least two time points available.

#### 2.1.2 CRYO Study

CRYO was a case–control lifestyle intervention study [24, 25] with 19 adults with obesity but without type 2 diabetes or medications. Participants underwent a 6‐week low‐calorie diet, followed by a weight‐maintenance phase with group and individual counseling focused on eating, physical activity, and behavioral therapy. Follow‐up measurements were done at 5 and 12 months.

Both studies followed the Declaration of Helsinki principles and received approval from the Ethics Committee of the Helsinki and Uusimaa Health District. Participants gave informed consent.

### 2.2 Anthropometrics, Body Composition and Physical Activity

Weight and height were measured in a fasting state with light clothing. Body composition was measured using dual‐energy X‐ray absorptiometry (Lunar iDXA, GE Healthcare, Wisconsin, USA) and analyzed using enCORE Software Platform v17 (GE Healthcare). Physical activity was measured using the Baecke questionnaire [26].

### 2.3 Clinical Chemistry

Blood samples were collected after a 12‐h fast and frozen at −80°C. Samples were analyzed at HUSLAB (Helsinki University Hospital, Finland) for blood count, lipids, glucose homeostasis, and high‐sensitivity C‐reactive protein (hs‐CRP) using standardized methods [7, 23].

### 2.4 Skeletal Muscle Biopsy Collection

After fasting blood sampling, a needle biopsy was taken from the vastus lateralis muscle under sterile conditions and local anesthesia with a 5‐mm Bergström needle [27]. The muscle specimen was divided, with samples for DNA and protein extractions snap‐frozen in liquid nitrogen and stored at −80°C. One piece was collected for transmission electron microscopy (TEM) and, in the surgery cohort, another was preserved for ex vivo mitochondrial respiration.

### 2.5 Sample Preparations for Proteomics

Proteins were extracted from the snap‐frozen muscle specimens in the lifestyle cohort (*n* = 19) and a subset of the surgery cohort (*n* = 33). Briefly, approximately 15 mg of tissue was homogenized in a RIPA‐M buffer, followed by a full lyse in 8 M urea. The proteins were precipitated by acetone and further processed using a commercial kit (PreOmics, Germany). Peptide digestion, washing, and preparation to 0.5 g/L concentration in “LC‐LOAD” solvent were performed according to manufacturer's instructions (Data S1: Methods).

### 2.6 Proteomics Analyses

LC–MS/MS was performed using a Q Exactive Hybrid Quadrupole‐Orbitrap Mass Spectrometer (Thermo Scientific) connected to an Ultimate 3000 RSLCnano (Dionex) ultra‐high pressure nanoflow chromatography system. Peptides were separated on an in‐house C18 column (150 nm × 0.075 mm × 3 μm, Dr. Maisch Reprosil‐Pur) over 120 min at a flow rate of 250 nL/min with a linear gradient of acetonitrile increasing from 1% to 27%. The mass spectrometer was operated in data dependent mode (70 000 FWHM, 300–1600 m/z), selecting the 12 most intense ions for fragmentation (Data S1: Methods).

MS/MS spectra were matched against Uniprot *Homo sapiens* database (2021_03) containing 78 120 entries using MaxQuant (version 2.0.3.0) [28, 29]. Label‐free quantitative ion intensities were generated by specifying trypsin as the digestion enzyme whilst allowing two missed cleavages and a 1% false discovery rate on peptides and proteins in searches (Data S1: Methods).

### 2.7 Transmission Electron Microscopy (TEM)

Samples for TEM analyses were fixed in 2.5% glutaraldehyde. For plastic embedding, they were treated with 1% osmium tetroxide, dehydrated in ethanol, and embedded in epoxy resin. Sections were stained with methylene blue (0.5%, w/v) and boric acid (1%, w/v), then examined with a light microscope to select areas for TEM. 60–90 nm sections were cut and stained with uranyl acetate and lead citrate, and examined with a JEM‐1400 microscope (Jeol).

From a subset (surgery cohort, *n* = 17; lifestyle cohort, *n* = 8), 15 TEM images were taken at each timepoint from the intermyofibrillar area. Participants were selected based on weight loss outcomes: individuals without (*n* = 9) and with (*n* = 8) type 2 diabetes with average weight loss were chosen from the surgery cohort, while those with the greatest weight loss were selected from the lifestyle cohort (*n* = 8) in an effort to match the degree of weight loss observed in the surgery cohort. Each single mitochondrion was painted in Microscopy Image Browser [30], and morphological characteristics were analyzed. Moreover, lipid droplets were painted and counted (See Data S1: Methods).

### 2.8 mtDNA Amount Quantification

DNA was extracted using 20–30 mg of frozen skeletal muscle specimen with the AllPrep RNA, DNA, miRNA Universal Kit (QIAGEN, Nordic, Solletuna, Sweden). The qPCR was performed with 10 ng of total DNA using SYBR Green PCR Master Mix (iQ Custom SYBR Green Supermix; Bio‐Rad). *MT‐CYTB* and *MT‐ND5* were used as mitochondrial and *HBB* and *B2M* as nuclear genes (Data S1: Methods). Data were analyzed in qBASEplus version 3.0 (Biogazelle).

### 2.9 High‐Resolution Respirometry

Fresh muscle specimen stored in BIOPS buffer (2.77 mM CaK2EGTA, 7.23 mM K2EGTA, 20 mM imidazole, 20 mM taurine, 50 mM MES hydrate, 0.5 mM DTT, 6.56 mM MgCl2, 5.77 mM ATP and 15 mM phosphocreatine) [31] was separated into fibers and treated with saponin (50 μg/mL) for 30 min and then washed with MiR05 buffer (0.5 mM EGTA, 3 mM MgCl_2_, 60 mM lactobionic acid, 20 mM taurine, 10 mM KH_2_PO_4_, 20 mM HEPES, 110 mM D‐sucrose, 1 g/L BSA). 1–3 mg of permeabilized fibers were measured in a respirometric chamber containing 2 mL MIR05 under ambient oxygen conditions with Oxygraph‐2 k equipment (O2k, Oroboros Instruments, Innsbruck, Austria). All measurements were achieved at the normal ambient oxygen range in the chamber and oxygen concentrations were above 100 nmol ml^−1^. All the used buffers, substrates and inhibitors were prepared according to Oroboros Instruments instructions [31].

The substrate‐uncoupler‐inhibition‐titration protocol began with background respiration measurement until a stable flux was achieved, usually around 5 min. Complex I (CI)‐mediated leak respiration was assessed with sodium glutamate (10 mM), sodium malate (2 mM), and sodium pyruvate (5 mM), followed by CI‐mediated respiration with 5 mM ADP. Mitochondrial membrane integrity check was checked by 10 μM cytochrome c. Maximal ADP‐stimulated coupled respiration was measured by convergent input of electrons via CI and complex II (CII) after adding 10 mM succinate. Oligomycin (10 μM), an ATP synthase inhibitor, determined CI & CII‐mediated leak respiration. FCCP (2 to 2.5 μM) was stepwise titrated until maximal uncoupled respiration (max CI & CII) was reached. 0.5 μM rotenone was added to inhibit CI and obtain maximal CII respiration. Non‐mitochondrial respiration was determined with 2.5 μM antimycin A as it inhibits complex III (CIII), the level of which was subtracted from all other values as background.

Oxygen flux was quantified using the DatLab analysis software 7.3.0.3 and corrected for muscle wet mass and given as oxygen flux expressed as pmol mg^−1^ s^−1^. All traces met the cytochrome c quality control criteria. We also calculated coupling efficiency (CoupEff): (CI & CII—Leak CI & CII)/CI & CII, indicating the proportion of ATP synthesis‐linked respiration from CI & CII respiration, and coupling control ratio (L/P): Leak CI/CI & CII, indicating the proportion of CI‐linked proton leak from CI & CII respiration.

### 2.10 Statistical Analyses

All data were analyzed using SPSS for Mac (version 29.0.2.0; SPSS Inc., Chicago, IL, USA) or R (version 4.3.2) and RStudio (2023.9.1.494). Figure and table legends specify statistical tests, sample sizes, and significance thresholds. Data are reported as mean ± SD or median [IQR] for skewed variables.

#### 2.10.1 Anthropometric and Clinical Variables

Anthropometric and clinical changes were analyzed using a linear mixed model with time point as explanatory variable with Restricted Maximum Likelihood (REML). Bonferroni correction adjusted the timepoint comparisons. Skewed variables were visually inspected by histograms and Q–Q plots and log_e_‐transformed before analysis. We considered *p* < 0.05 statistically significant.

#### 2.10.2 Proteomics Data Processing and Differential Expression Analysis

In skeletal muscle proteomics analyses, we selected only those proteins with valid identifications and filtered out proteins identified exclusively by peptides carrying one or more modified amino acids, as well as decoy proteins marked as reverse, to ensure data quality. We log_2_‐transformed the data and applied conservative filtering, retaining only those proteins detected in at least six (30%) samples at any time point in any group. We imputed the missing values using the *impute_na* function with the minProb method (promor package) [32], applying a Gaussian distribution centered on each protein's minimal observed value [33].

Differential protein analysis used dream (VariancePartition package) [34] with linear mixed models including group, weight loss percentage (modeled continuously to estimate change per %‐point), and their interaction as fixed effects, and a random intercept per participant. Baseline weight and height were covariates. The data was split into baseline–5/6‐month and baseline–12‐month subsets, and models were fit separately for each. To assess whether baseline type 2 diabetes status was associated with protein changes in the surgery cohort, group was replaced with diabetes status. Proteome baseline differences were derived from the same models but using time point as an explanatory variable.

#### 2.10.3 Biological Pathway Analyses

We investigated significantly changed proteins (surgery cohort FDR *p* < 0.05; lifestyle cohort *p* < 0.05) using Ingenuity Pathway Analysis (IPA) with *p* < 0.05 as cutoff (version 145 030 503, Ingenuity Systems, Redwood City, CA, USA). The Ingenuity Knowledge Base was used as the reference set.

### 2.11 Mitochondrial Measures

Changes in targeted mitochondrial parameters (e.g., mtDNA amount, TEM, and high‐resolution respirometry) were analyzed using a generalized linear model with time point as explanatory variable and Bonferroni correction. We considered *p* < 0.05 statistically significant. Spearman's rank correlations were used to evaluate associations between changes in anthropometric/clinical parameters and changes in mitochondrial markers from baseline to 12 months.

## 3 Results

### 3.1 Participant Characteristics at Baseline and After Weight Loss

At baseline, the participants from the surgery cohort, compared to the participants in the lifestyle cohort, were older, had more body adiposity and more appendicular soft lean tissue mass, and were metabolically unhealthier with regards to glucose metabolism and inflammation (Table 1). The participants who underwent surgery also scored lower on the Baecke sports index at baseline (Table 1).

**TABLE 1:** Participant characteristics.

	Lifestyle cohort (*n* = 19)				Bariatric surgery cohort (*n* = 39)				*p*, baseline
	Baseline	5 months	12 months	*p*	Baseline	6 months	12 months	*p*	
Sex (females %)	63.2				66.7				1.000
Age (years)	35.8 ± 7.7				46.8 ± 7.4				**< 0.001**
Body weight (kg)	99.0 ± 14.0	87.4 ± 14.2*	90.1 ± 15.0	**0.042**	131.1 ± 19.4	103.7 ± 15.6*	99.0 ± 16.4*	**< 0.001**	**< 0.001**
Waist (cm)	112.5 ± 10.1	100.1 ± 11.3*	99.4 ± 11.8*	**< 0.001**	133.6 ± 13.6	112.3 ± 10.3*	107.4 ± 11.7*	**< 0.001**	**< 0.001**
BMI (kg/m^2^)	34.7 ± 2.7	30.6 ± 3.4*	31.6 ± 3.9*	**0.001**	45.3 ± 5.7	35.9 ± 4.9*	34.2 ± 5.2*	**< 0.001**	**< 0.001**
Body fat (%)	48.8 [39.4: 51.7]	43.8 [34.9: 46.9]	46.1 [33.9: 49.8]	0.188	50.9 [45.8: 53.6]	41.4 [35.9: 46.4]	38.2 [31.2: 45.8]	**< 0.001**	0.094
Body fat (kg)	43.7 ± 7.9	34.8 ± 10.1*	36.7 ± 11.5	**0.019**	64.0 ± 13.1	42.6 ± 11.1*	37.4 ± 11.9*	**< 0.001**	**< 0.001**
Lean soft tissue mass (kg)	50.5 [41.5: 64.6]	47.2 [41.6: 59]	47.7 [40.8: 61.8]	0.800	59.7 [55.7: 68.5]	55.5 [51: 63]	55.1 [50.2: 62.2]*	**0.020**	**< 0.001**
Appendicular lean soft tissue mass (kg)	23.7 ± 6.1	22.4 ± 5.4	22.4 ± 5.5	0.725	28.5 ± 5.5	25.9 ± 4.4	25.2 ± 5.0*	**0.012**	**0.004**
Appendicular lean mass index	8.2 ± 1.4	7.7 ± 1.1	7.8 ± 1.1	0.437	9.8 ± 1.3	8.9 ± 1.0*	8.7 ± 1.1*	**< 0.001**	**0.001**
Fasting glucose (mmol/L)	5.7 [5.3: 6.1]	5.3 [5.2: 5.8]	5.5 [5: 5.8]	0.190	6.3 [5.6: 6.9]	5.5 [5.1: 5.8]*	5.4 [5.1: 5.8]*	**< 0.001**	**0.004**
Fasting insulin (mU/L)	8.1 [5.8: 12.9]	7.3 [4.7: 10.7]	6.8 [5.8: 11.3]	0.511	16.6 [11.2: 24.5]	9 [5.3: 12]*	6.2 [4.7: 9.4]*	**< 0.001**	**0.025**
HOMA‐IR index	2.0 [1.5: 3.3]	1.9 [1: 2.5]	1.6 [1.3: 3.2]	0.402	4.3 [3.1: 7.3]	2.3 [1.3: 3]*	1.6 [1.2: 2.4]*	**< 0.001**	**0.031**
Matsuda index	4.5 [2.7: 6.2]	6.6 [4.7: 11]	5.9 [4.7: 8.1]	0.161	2.2 [1.3: 3.7]	4 [2.7: 5.2]*	5.3 [3.3: 8.2]*	**< 0.001**	**< 0.001**
Total cholesterol (mmol/L)	4.6 ± 0.7	4.2 ± 0.7	4.4 ± 0.7	0.164	4.5 ± 0.9	3.8 ± 0.7*	4.0 ± 1.0	**0.003**	0.591
HDL (mmol/L)	1.4 ± 0.3	1.4 ± 0.2	1.5 ± 0.3	0.278	1.2 ± 0.2	1.2 ± 0.2	1.4 ± 0.3*	**0.009**	0.053
LDL (mmol/L)	2.9 ± 0.6	2.5 ± 0.6	2.6 ± 0.6	0.154	2.8 ± 0.8	2.2 ± 0.7*	2.3 ± 0.8*	**0.001**	0.733
TAG (mmol/L)	1.0 [0.8: 1.6]	0.7 [0.5: 1.0]*	0.8 [0.6: 1.0]	**0.006**	1.4 [1.0: 1.8]	1.2 [0.8: 1.3]*	0.9 [0.8: 1.1]*	**< 0.001**	0.060
Work index	2.6 ± 0.7	2.7 ± 0.8	2.8 ± 0.7	0.742	2.5 ± 0.6	2.5 ± 0.6	2.6 ± 0.6	0.916	0.694
Sports index	2.5 ± 0.5	3.0 ± 0.7*	2.8 ± 0.7	**0.045**	2.0 ± 0.7	2.4 ± 0.9	2.5 ± 0.9	**0.044**	**0.016**
Leisure index	2.8 ± 0.6	3.3 ± 0.7	3.2 ± 0.7	0.096	2.7 ± 0.6	2.9 ± 0.6	3.0 ± 0.5	0.074	0.408
Total physical activity (Baecke)	7.9 ± 1.3	9.1 ± 1.3*	8.9 ± 1.3	**0.030**	7.4 ± 1.3	8.1 ± 1.3	8.3 ± 1.3*	**0.034**	0.191
CRP (mg/L)					5.8 ± 5.2	2.2 ± 2.6*	1.2 ± 1.1*	**< 0.001**	
Leukocytes	5.8 ± 1.2	5.8 ± 1.2	5.5 ± 1.0	0.620	6.7 ± 1.4	5.8 ± 1.2*	5.5 ± 1.4*	**< 0.001**	**0.032**

*Note:* Data are reported as mean ± SD (normally distributed variables) or median (interquartile range for skewed variables). *p* values were obtained using a linear mixed model using the REML method to study the effects of follwup time point on anthropometric and clinical parameters before and after weight loss intervention. We also employed the Bonferroni post hoc test; *p < 0.05 different from baseline. *p* values for baseline differences between lifestyle and surgery cohorts were obtained using an independent samples *t*‐test. We considered *p* < 0.05 significant and bolded these values. Skewed variables were log_e_‐transformed before analysis.

Abbreviations: BMI, body mass index; CRP, C‐reactive protein; HDL, high‐density lipoprotein; HOMA‐IR, homeostatic model for the assessment of insulin resistance; LDL, low‐density lipoprotein; TAG: triacylglycerol.

In the surgery cohort, average weight loss reached 20.8% ± 5.0% at 6 months and 24.4% ± 7.3% at 12 months. In the lifestyle cohort, participants lost on average 11.8% ± 5.3% at 5 months and 9.0% ± 7.4% at 12 months. Clinical measures for lipid metabolism and insulin sensitivity generally reflected changes in weight and were improved in both cohorts, particularly following bariatric surgery. The total physical activity and sports indexes improved in both cohorts (Table 1). In the surgery cohort, changes over time were not statistically different between participants with and without type 2 diabetes (Table S1).

### 3.2 Skeletal Muscle Proteome Differences Between Cohorts at Baseline

We first explored baseline differences in the global proteome, covering 1050 proteins, between the two weight loss cohorts. At baseline, 103 proteins showed differential expression (FDR *p* < 0.05), with 60% downregulated in the surgery cohort compared to the lifestyle cohort (Table S2). The top pathways were associated with downregulated 40S ribosomal subunit proteins and downregulated respiratory electron transport proteins in the surgery cohort compared to the lifestyle cohort (Table S3), suggesting lower baseline protein synthesis and mitochondria‐related protein levels in the surgery cohort.

### 3.3 The Skeletal Muscle Proteome Alterations Following Surgery and Lifestyle Intervention

Six months after surgery, we identified 47 significantly (FDR *p* < 0.05) altered proteins that associated with weight loss percentage, with 55% showing downregulation compared to baseline. At 12 months, 57 proteins were significantly associated with weight loss percentage, of which 81% were downregulated compared to baseline (Table S2). In the lifestyle cohort, none of the proteins were associated with weight loss percentage (FDR *p* < 0.05) at 5 or 12 months. Although adjusted *p*‐values were not significant, clear signals emerged that merited further investigation. At 5 months we identified 104 proteins associating with weight loss percentage (nominal *p* < 0.05), with 44% showing downregulation. At 12 months, 43 proteins were significantly associated, with 67% downregulated (Table S2). These findings indicate that surgery is associated with more pronounced and sustained proteomic remodeling in skeletal muscle compared to a lifestyle intervention.

### 3.4 Downregulation of Cholesterol Signaling Proteins at 6‐Months and Glycolysis Proteins at 12‐Months Post‐Surgery

**FIGURE 1:**
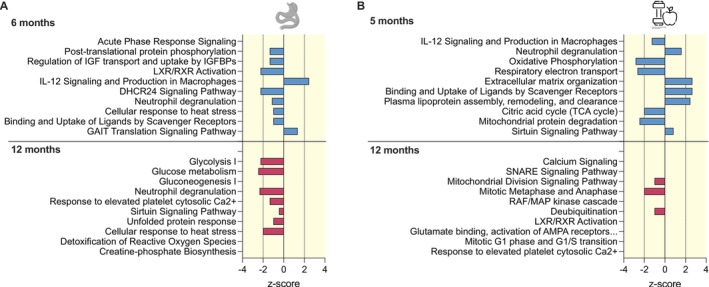
Top 10 biological pathways for skeletal muscle proteome changes upon surgery‐induced and lifestyle‐induced weight loss interventions. Protein enrichment for biological pathways using differentially expressed proteins associated with weight loss percentage at the respective timepoint in the surgery cohort (RYSA, *n* = 33) (FDR *p* < 0.05) (A) and lifestyle cohort (CRYO, *n* = 19) (nominal *p* < 0.05) (B). The top 10 significant findings from the ingenuity pathway analysis (IPA) tool (*p* < 0.05) are shown. IPA calculated pathway directionality *z*‐scores based on the number of activated (*z*‐score > 0), inhibited (*z*‐score < 0), or neutral (*z*‐score = 0) genes. *Z*‐scores > 2 or < −2 were considered statistically significant. Results ranked according to statistical significance.

Six months after surgery, weight loss percentage was primarily associated with cholesterol signaling (e.g., LXR/RXR Activation, *z*‐score: −2.23) and acute phase response signaling pathways (Figure 1A). The downregulated proteins included APOB, C3, and ORM1/ORM2. At 12 months, the top changed pathway was glycolysis (*z*‐score: −2.24) with downregulated proteins like ALDOA, ENO3, GAPDH, PGAM2, PKM, and TPI1 (FDR *p* < 0.05) (Figure 1A, Table S2). Moreover, detoxification of ROS (e.g., downregulated PRDX1 and SOD1 proteins) and unfolded protein response pathways were enriched (Figure 1A, Table S4).

### 3.5 Downregulation of Oxidative Phosphorylation Proteins at 5‐Months in the Lifestyle Cohort

In the lifestyle cohort, at 5 months, weight loss percentage was related to mitochondrial function pathways, which were predicted to be downregulated, including OXPHOS (*z*‐score: −2.83) and TCA cycle (*z*‐score: −2.00) (Figure 1B). At 12 months, proteins related to calcium signaling were overrepresented, but without directionality available (Figure 1B, Table S5).

### 3.6 Mitochondria‐Related Protein Changes Following Both Weight Loss Interventions

Since mitochondrial function is crucial for energy metabolism and skeletal muscle adaptation to weight loss [10], we focused on mitochondria‐related proteins from the MitoMiner database [35]. Of 2210 MitoMiner proteins, 391 were detected (Table S2). After surgery, the top MitoMiner protein pathways were related to protein unfolding and BCAA catabolism at 6 months, and glycolysis and detoxification of ROS at 12 months (Figure S1A). In the lifestyle cohort, at 5 months, the top pathways were related to OXPHOS and at 12 months to fatty acid activation (Figure S1B). However, the directionality of these pathways could not be determined, except for downregulation of OXPHOS at 5 months in the lifestyle cohort (*z*‐score: −2.7).

### 3.7 Skeletal Muscle Mitochondrial Number, Shape and Lipid Droplets Remained the Same Following Both Weight Loss Interventions

**FIGURE 2:**
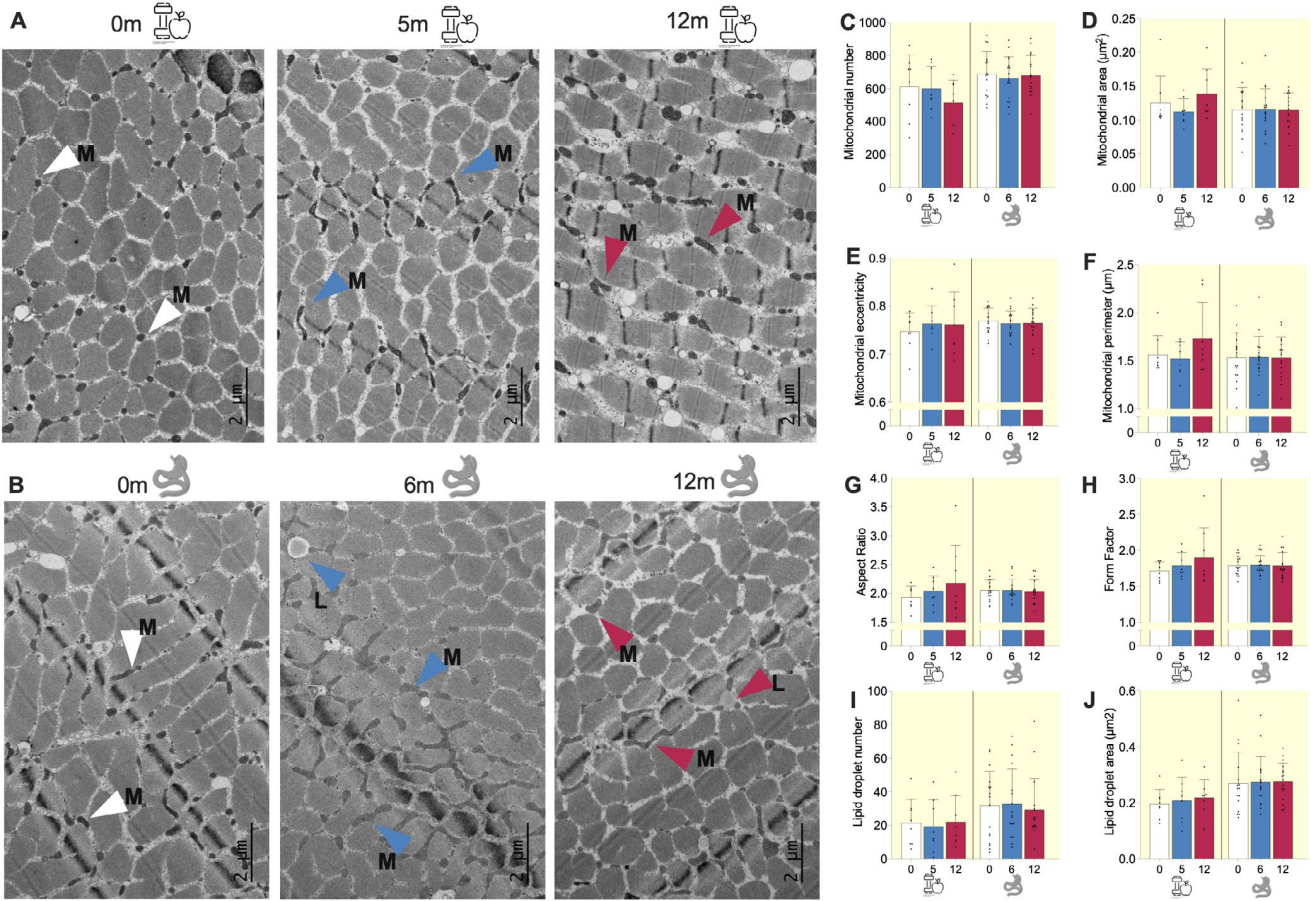
Skeletal muscle mitochondrial and lipid droplets number and morphology did not significantly change following surgery‐ and lifestyle‐induced weight loss interventions. TEM images of muscle intermyofibrillar mitochondria (M) and lipid droplets (L) from two representative study participants before and after (A) lifestyle‐induced weight loss and (B) and after surgery‐induced weight loss. Magnification, ×2000. Scale bars, 2 μm. Quantification of (C) the number of mitochondria, (D) mitochondrial surface area per total muscle fiber area, (E) average eccentricity of mitochondria, (F) average perimeter of mitochondria, (G) aspect ratio (the length‐to‐width ratio of mitochondria), (H) form factor (the branching of mitochondria), (I) lipid droplet number and (J) average lipid droplet area in skeletal muscle. The bars indicate the mean ± SD and dots indicate individual measurements. Blue bars: lifestyle cohort (*n* = 8), red bars: surgery cohort (*n* = 17).

To assess potential structural mitochondrial changes following weight loss, we conducted a quantitative analysis using TEM (Figure 2A,B), measuring mitochondrial number and area (mass) (Figure 2C,D), perimeter, eccentricity (roundness), form factor (branching), and aspect ratio (length‐to‐width ratio) (Figure 2E–H) in a subset of participants. Mitochondrial morphology and spatial organization are key indicators of functional integrity, with fragmentation often linked to dysfunction and increased branching associated with healthier mitochondria [36]. Overall, none of these parameters changed significantly after weight loss, regardless of whether it was induced by surgery or a lifestyle intervention.

Moreover, considering the close interplay between mitochondria and lipid storage, along with studies reporting lipid droplet remodeling following weight loss [37], we next analyzed lipid droplet number and area after both interventions. We found that the number and area of lipid droplets remained statistically unchanged after weight loss, whether achieved through surgery or lifestyle intervention (Figure 2I,J).

### 3.8 MtDNA Amount Decreased Following Weight Loss

**FIGURE 3:**
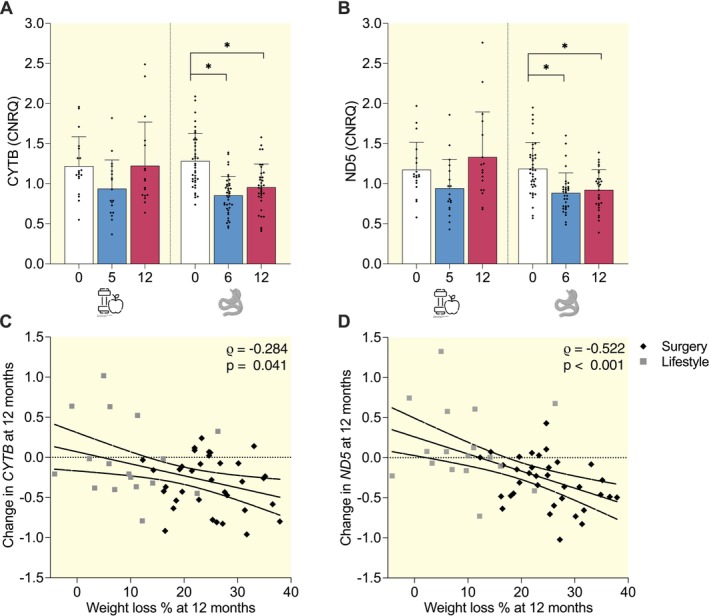
Skeletal muscle mtDNA amount following surgery‐ and lifestyle‐induced weight loss. Relative mtDNA amount to nuclear DNA content measured by amplifying (A) *CYTB* and (B) *ND5* mitochondrial gene areas with qPCR. The bars indicate the mean ± SD and dots indicate individual measurements. Blue bars: lifestyle cohort (CRYO, *n* = 19), red bars: surgery cohort (RYSA, *n* = 39). *p < 0.05 (generalized linear model with time point as explanatory variable and Bonferroni post hoc correction). (C) and (D) Spearman rank correlations of weight loss percentage with change in (C) CYTB and (D) ND5 from baseline to 12 months.

MtDNA amount, a marker for mitochondrial content and biogenesis, decreased post‐surgery at both 6‐months (e.g., *CYTB* Fold Change (FC) 0.70 ± 0.23 AU, *p* < 0.001) and 12‐months (*CYTB* FC 0.77 ± 0.23 AU, *p* < 0.001). In the lifestyle cohort, mtDNA amount was not statistically changed (e.g., *CYTB* FC 6‐months: 0.80 ± 0.27 AU; FC 12‐months: 1.02 ± 0.43 AU) (Figure 3). Pooling both cohorts to span the full weight‐loss range, weight loss percentage correlated negatively with mtDNA change at 12‐months (CYTB *ρ* = −0.284, *p* = 0.041; ND5 *ρ* = −0.522, *p* < 0.001; *n* = 52; Figure 3C,D; Table S6), indicating that greater weight loss, irrespective of intervention type, is associated with lower mtDNA.

### 3.9 Skeletal Muscle Mitochondrial Respiration Increased Following Surgery

**FIGURE 4:**
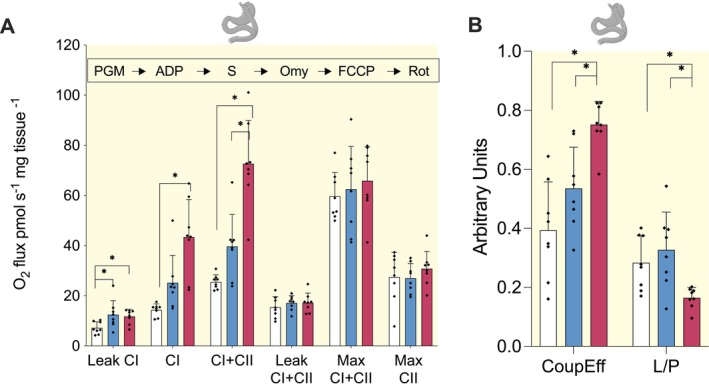
Mitochondrial respiration increases following surgery‐induced weight loss. (A) Mitochondrial respiration normalized to tissue mass. (B) Coupling efficiency (CoupEff) and LEAK/OXPHOS (L/P) ratio normalized to tissue mass. Red bars: surgery cohort (*n* = 8). Data are mean ± SD and dots indicate individual measurements. *p < 0.05 (generalized linear model with time point as explanatory variable and Bonferroni post hoc correction). Added substrates and inhibitors for respiration measurements are shown in chronological order over the plot. FCCP, carbonylcyanide‐4‐(trifluoromethoxy)‐phenylhydrazone; Omy, oligomycin; PGM, pyruvate, glutamate, malate; Rot, rotenone; S, succinate.

To investigate potential functional improvements in mitochondrial respiration after surgery, we performed high‐resolution respirometry. At 12 months, compared to baseline, CI‐mediated respiration increased 3.2‐fold (43.4 ± 15.0 vs 14.3 ± 2.4 pmol mg^−1^ s^−1^, *p* < 0.001) and CI + II‐mediated respiration increased 2.9‐fold (72.7 ± 17.2 vs 25.5 ± 2.9, *p* < 0.001). Leak CI‐mediated respiration increased 2.1‐fold at 6 months (*p* < 0.011) and 1.8‐fold at 12 months (*p* < 0.037) (Figure 4A). Maximal uncoupled CI + II‐ and CII‐mediated respiration and CI + II‐mediated leak respiration remained similar. The coupling efficiency (i.e., the proportion of ATP synthesis‐linked respiration from CI & CII respiration) increased at 12‐months (*p* < 0.001), indicating improved mitochondrial efficiency (Figure 4B). In line, the coupling control (*L/P*) ratio (i.e., the proton leak relative to total proton gradient generated by CI + CII) decreased significantly after 12 months (Figure 4B) and the CI + CII‐mediated coupled respiration nearly matched the uncoupled maximal CI + II‐mediated respiration at 12 months, indicating an increased coupling capacity (Figure 4A). This implies that after bariatric surgery, muscle utilizes the OXPHOS machinery more efficiently for energy conversion.

Physical activity is a strong regulator of mitochondrial function [11]. We found a positive correlation between increases in Baecke sports index and those in CI‐mediated respiration between baseline and 12 months (*ρ* = 0.82, *p* = 0.046, *n* = 6, Table S6), indicating that physical activity may relate to improved mitochondrial function.

### 3.10 Skeletal Muscle Proteome, but Not Other Mitochondrial Markers, Responded Differently in People With Type 2 Diabetes Following Surgery

Within the surgery cohort, we performed a sub‐analysis to assess the link between type 2 diabetes status and mitochondrial measures [38]. At baseline, no proteins differed between non‐diabetes and diabetes at the FDR *p* < 0.05 level, but 94 proteins showed nominal difference (*p* < 0.05) (Table S7), with upregulated OXPHOS and electron transport pathways in the individuals with diabetes (*z* > 2.4) (Table S8).

At 6 months, 20 proteins (FDR *p* < 0.05) associated with weight loss percentage in individuals without diabetes, but none in those with diabetes. In individuals without diabetes, top pathways for proteins associating with weight loss percentage (nominal *p* < 0.05) included increased OXPHOS and mitochondrial protein degradation (*z*‐scores > 4), whereas in those with diabetes weight loss percentage associated with downregulated OXPHOS (*z*‐score: −3.3) and beta‐oxidation at 12 months (Figure S2, Table S9). Mitochondrial morphology (Figure S3), mtDNA amount and respiration (Figure S4) responses to surgery showed no diabetes‐specific differences. These results indicate that metabolic status may modulate skeletal muscle mitochondrial protein responses to surgery, while other mitochondrial features appear similar.

## 4 Discussion

In this study, we offer new insights into how weight loss from bariatric surgery or a lifestyle intervention affects skeletal muscle mitochondria. Both interventions led to clinically meaningful weight loss, but their impact on mitochondrial markers and the muscle proteome varied. Following bariatric surgery, skeletal muscle mitochondrial efficiency improved as respiration was increased, despite a reduction in mtDNA amount, and stable mitochondrial number, morphology, and mitochondria‐related protein expression. Diabetes status may have modulated mitochondria‐related protein expression to surgery. OXPHOS complex subunit proteins were associated with upregulation in Individuals without diabetes but downregulation in those with diabetes. In contrast, after lifestyle‐induced weight loss, we observed a decrease in OXPHOS complex subunit proteins at 5 months, while other mitochondrial markers remained stable.

Our key finding was that surgery‐induced weight loss was associated with increased ADP‐stimulated coupled mitochondrial respiration. Prior studies on the effects of bariatric surgery and skeletal muscle mitochondrial respiration have been inconsistent [10]. While some report increased Complex I‐linked ADP‐stimulated respiration [14], others describe unchanged or reduced mitochondrial respiration after RYGB [15, 16]. We observed a greater coupling efficiency and lower coupling control (L/P) ratio, indicating improved mitochondrial efficiency and improved ATP production relative to proton leak. This pattern indicates bioenergetic efficiency gains without detectable changes in mitochondrial content or morphology [39]. Supporting this, ROS detoxification proteins were downregulated after surgery, consistent with reduced oxidative stress. Additionally, decreased glycolysis‐related protein expression supports enhanced mitochondrial respiration, indicating a metabolic shift toward more oxidative metabolism [40]. Overall, our findings indicate that bariatric surgery may improve skeletal muscle mitochondrial efficiency and oxidative capacity.

Mitochondrial biogenesis markers, for example, mitochondrial number and morphology, were not substantially changed in either weight‐loss cohort. The impact of obesity on skeletal muscle mitochondrial morphology varies, with some studies showing improvements in subsarcolemmal mitochondria and others in the intermyofibrillar fraction upon nutritional or exercise stimuli [6]. Our analysis focused on intermyofibrillar mitochondria, leaving other muscle fiber subtypes or regions near the sarcolemma unexplored. MtDNA amount is often used as a marker of mitochondrial biogenesis, but it has shown a poor correlation with mitochondrial number/content, mass, and activity in skeletal muscle [41]. While mtDNA levels decreased after surgery, a stable mitochondrial number and morphology suggest this does not reflect mitochondrial content loss but may indicate altered mtDNA regulation in mtDNA replication, repair, or degradation. This mtDNA amount reduction may be linked to improved mitochondrial efficiency, where lower mtDNA copy number suffices due to enhanced mitochondrial function. Few mitochondria‐related proteins were downregulated post‐surgery, mainly in ROS detoxification pathways, implying reduced oxidative stress rather than impaired mitochondrial biogenesis. In the lifestyle cohort, OXPHOS‐related protein expression decreased at 5 months, with no other statistically significant changes in mitochondrial biogenesis markers, consistent with previous findings of minimal mitochondrial adaptation to lifestyle‐induced weight loss [10]. Overall, our findings indicate that post‐surgery mitochondrial improvements may be qualitative rather than quantitative, with enhanced respiration and minimal or no statistically significant changes in mitochondrial biogenesis markers.

Neither surgery‐induced nor lifestyle‐induced weight loss altered myocellular lipid droplet number or area. Previous studies have reported reduced lipid droplets following bariatric surgery, with decreased intramuscle triglyceride content after RYGB [11, 12] and biliopancreatic diversion [42], at varying intervals from 3 to 18 months. Results on lipid droplets following lifestyle‐induced weight loss are less consistent. After 7 weeks of very low caloric diet, no changes in intramuscular triglycerides were observed [43], but their levels were increased after 16‐week caloric restriction [44]. Further studies are needed to define how weight loss modifies intramyocellular lipid droplet dynamics and composition, and how they affect mitochondrial metabolism [37].

We also examined whether baseline diabetes status affected mitochondrial adaptations after surgery. For instance, in a prior RYGB study, females with diabetes showed downregulated OXPHOS genes preoperatively but upregulated post‐surgery in parallel with improved glucose metabolism, whereas individuals without diabetes showed no change [45]. At baseline, we observed higher expression of some OXPHOS complex subunit proteins, contrary to other studies [46, 47]. Post‐surgery, we found increased skeletal muscle OXPHOS protein expression in individuals without diabetes, but downregulation in those with diabetes, despite no apparent changes in mitochondrial morphology or mtDNA amount. These findings indicate that diabetes status may modulate OXPHOS complex subunit proteins' responses to surgery.

Overall, this study suggests that skeletal muscle mitochondrial metabolism upon weight loss may be affected differently by surgery and lifestyle intervention. While the mechanisms remain unclear, bariatric surgery may enhance mitochondrial oxidative metabolism by multiple signals such as bile acids [48], gut hormones [49], and microbiome‐related products including short‐chain fatty acids [50], which also respond differently to weight‐loss methods. Physical activity stimulates skeletal muscle mitochondrial biogenesis and oxidative metabolism [51] and combining a low‐calorie diet with exercise is more effective [10]. Correspondingly, we observed a positive correlation between changes in physical activity and changes in CI‐mediated respiration.

The primary strength of this study is having a uniform set of high‐quality mitochondrial analyses (proteomics, TEM, mtDNA amount) across both weight loss cohorts, allowing comparison at similar timepoints while minimizing technical variability. Although energy balance may have varied during biopsies, participants likely achieved stable weight at 12 months. However, differences between cohorts, including age, presence of type 2 diabetes, and the magnitude of weight loss may influence mitochondrial outcomes and should be considered when interpreting the results. Mitochondrial and lipid droplet characteristics can vary by muscle fiber type, but TEM imaging did not allow fiber‐type differentiation. Additionally, we only examined mitochondrial respiration following bariatric surgery. Moreover, we did not measure muscle strength and endurance in either cohort, which limits interpretation of how the observed mitochondrial adaptations relate to muscle function. Future studies should explore the functional consequences of lifestyle‐induced weight loss on skeletal muscle mitochondrial activity and function as posttranscriptional and/or translational regulatory mechanisms may be important.

In summary, after bariatric surgery, skeletal muscle mitochondrial efficiency improved as respiration was increased, despite a reduction in mtDNA amount, and stable mitochondrial number, morphology, and mitochondria‐related protein expression. After lifestyle‐induced weight loss, OXPHOS proteins declined at the 5‐month mark, while other mitochondrial markers remained stable. Diabetes status affected mitochondria‐related proteins differentially, but not other mitochondrial markers. In skeletal muscle, weight loss through bariatric surgery may preserve mitochondrial capacity, but may be influenced by type 2 diabetes. Future investigations should prioritize exploring skeletal muscle oxidative capacity following lifestyle‐induced weight loss and different metabolic states.

## Author Contributions

**Birgitta W. van der Kolk:** conceptualization, investigation, data curation, formal analysis, methodology, visualization, writing – original draft, writing – review and editing, funding acquisition. **Sini Heinonen:** conceptualization, funding acquisition, investigation, methodology, writing – review and editing. **James W. White:** data curation, investigation, writing – review and editing. **Anita Wagner:** data curation, investigation, writing – review and editing. **Jari E. Karppinen:** data curation, formal analysis, methodology, writing – review and editing. **Sina Saari:** investigation, writing – review and editing. **Maheswary Muniandy:** methodology, writing – review and editing. **Simo Metsikkö:** investigation, writing – review and editing. **Eugène T. Dillon:** investigation, writing – review and editing. **Per‐Henrik Groop:** investigation, writing – review and editing. **Tuure Saarinen:** investigation, writing – review and editing. **Carel W. Le Roux:** investigation, writing – review and editing. **Kirsi A. Virtanen:** conceptualization, funding acquisition, investigation, resources, writing – review and editing. **Neil G. Docherty:** investigation, supervision, writing – review and editing. **Eija Pirinen:** funding acquisition, investigation, supervision, writing – review and editing. **Anne Juuti:** conceptualization, funding acquisition, investigation, resources, supervision, writing – review and editing. **Kirsi H. Pietiläinen:** conceptualization, data curation, funding acquisition, investigation, methodology, resources, supervision, writing – review and editing.

## Funding

The study was supported by the Research Council of Finland (272376, 266286, 314383, 335443, 369181 to Kirsi H. Pietiläinen; 314457 to Anne Juuti; 259926, 265204, 292839, 314456, 335446 to Kirsi A. Virtanen; 361956 and 338417 to Sini Heinonen; 335445, 314455, and Research Council of Finland Profi6 funding (336449) awarded to the University of Oulu: to Eija Pirinen); the Finnish Medical Foundation (Kirsi H. Pietiläinen, Sini Heinonen, Anne Juuti, Eija Pirinen, Kirsi A. Virtanen); the Finnish Diabetes Research Foundation (Birgitta van der Kolk, Sini Heinonen, Kirsi H. Pietiläinen, Kirsi A. Virtanen); the Orion Foundation Sr (Birgitta van der Kolk, Sini Heinonen); the Novo Nordisk Foundation (NNF10OC1013354, NNF17OC0027232, NNF20OC0060547, NNF24OC0091683 and NNF25SA0103783 to Kirsi H. Pietiläinen; NNF24SA0090438 to Birgitta van der Kolk; NNF23SA0083953 and NNF25OC0100827 to Sini Heinonen); the Paulo Foundation (Sini Heinonen, Kirsi H. Pietiläinen); the Gyllenberg Foundation (Kirsi H. Pietiläinen); the Finnish Foundation for Cardiovascular Research (Kirsi H. Pietiläinen); the Sigrid Juselius Foundation (Kirsi H. Pietiläinen, Kirsi A. Virtanen); the Paavo Nurmi Foundation (Sini Heinonen); Helsinki University Hospital Research Funds (Sini Heinonen, Kirsi H. Pietiläinen, and Anne Juuti); Government Research Funds (Kirsi H. Pietiläinen, Sini Heinonen, Kirsi A. Virtanen); and the University of Helsinki (Kirsi H. Pietiläinen).

## Conflicts of Interest

The authors declare no conflicts of interest.

## Supporting information


**Data S1:** apha70150‐sup‐0001‐suppl‐methods.docx.

**Table S1:** apha70150‐sup‐0002‐TableS1.docx.

**Table S2:** apha70150‐sup‐0003‐TableS2.xlsx.

**Table S3:** apha70150‐sup‐0004‐TableS3.xlsx.

**Table S4:** apha70150‐sup‐0005‐TableS4.xlsx.

**Table S5:** apha70150‐sup‐0006‐TableS5.xlsx.

**Table S6:** apha70150‐sup‐0007‐TableS6.xlsx.

**Table S7:** apha70150‐sup‐0008‐TableS7.xlsx.

**Table S8:** apha70150‐sup‐0009‐TableS8.xlsx.

**Table S9:** apha70150‐sup‐0010‐TableS9.xlsx.

**Figures S1–S4:** apha70150‐sup‐0012‐FiguresS1‐S4.zip.

## Data Availability

The mass spectrometry proteomics data have been deposited to the ProteomeXchange Consortium via the PRIDE [52] partner repository with the dataset identifier PXD061410.
