# Supplementary material for: Human Skeletal Muscle Mitochondria Responses to Weight Loss Induced by Bariatric Surgery or Lifestyle Intervention
Source: Acta Physiol (Oxf). 2026 Jan 8;242(2):e70150. doi: 10.1111/apha.70150 (PMC12783452; doi:10.1111/apha.70150)
Supplement: Supplementary file 1 — Data S1: apha70150‐sup‐0001‐suppl‐methods.docx. [file APHA-242-e70150-s004.docx]

**Human skeletal muscle mitochondria respond differently to weight loss induced by bariatric surgery or lifestyle intervention**

Birgitta W. van der Kolk^1^, Sini Heinonen^1,2^, James W. White^3^, Anita Wagner^1,4^, Jari E. Karppinen^1^, Sina Saari^1^, Maheswary Muniandy^1^, Simo Metsikkö^1^, Eugène T. Dillon^5^, Per-Henrik Groop^1,6-9^, Tuure Saarinen^10^, Carel W. Le Roux^3^, Kirsi A. Virtanen^11,12^, Neil G. Docherty^3^, Eija Pirinen^1,13-15^, Anne Juuti^3^, Kirsi H. Pietiläinen^1,16^

**Supplementary Methods**

***Sample preparation for LC-MS/MS analysis***

Proteins were extracted from the snap-frozen muscle specimens in the lifestyle cohort (n = 19) and a subset of the surgery cohort (n = 33). by homogenizing the tissue with a TissueLyser LT instrument (QIAGEN) in an ice-cold RIPA-M lysis buffer (150mM NaCl, 1mM EDTA, 50mM Tris, 1% IGE-PAL, pH 7.5) containing broad-range protease and phosphatase inhibitors. The homogenate was then centrifuged for 15 min at 20,000g at 4°C, and the supernatant was transferred to a new tube. The pellet was resuspended in UREA-T buffer (50mM Tris, 75mM NaCl, 8M Urea, pH 8.1) and sonicated for 5 min using a Bioruptor Pico. Homogenates were centrifuged and the supernatant was combined with the supernatant from the RIPA-M lysis buffer step in a new tube. Sample protein concentration was determined using Pierce BCA protein assay kit (Thermo Fisher Scientific).

Next, the proteins were precipitated by addition of 4x volume of ice-cold acetone and incubated for 1 hour at -20°C. After centrifugation (13,000g, 4 °C, 10min) the protein precipitant was further processed by using a commercial kit (PreOmics, Germany). Briefly, 50 μg protein of the samples were solubilized in ‘Lyse’ buffer and boiled at 95 °C for 10min. Then the samples were transferred to the cartridge and digested by adding 50 μl of the ‘Digest’ solution. After 60min of incubation at 37 °C the digestion was stopped with 100 μl of ‘Stop’ solution. The solutions in the cartridge were removed by centrifugation at 3′800g, while the peptides were retained by the iST-filter. Finally, the peptides were washed, eluted, dried, and re-solubilized in 50 μl ‘LC-Load’ solvent and stored at -80°C. Peptide samples were later prepared to 0.5 g/L concentration in ‘LC-LOAD’ solvent and transferred to glass mass spectrometry vials.

***LC-MS/MS analysis***

MS proteomics was performed by the Conway Institute Mass Spectrometry Core Facility at University College Dublin on a Q Exactive™ Hybrid Quadrupole-Orbitrap™ Mass Spectrometer (Thermo Scientific) connected to an Ultimate™ 3000 RSLCnano (Dionex) ultra-high pressure nanoflow chromatography system. Peptides were separated on an in-house C18 column (150 mm x 0.075 mm x 3 µm, C18-AQ Dr Maisch Reprosil-Pur) over 120 minutes at a flow rate of 250 nl/min with a linear gradient of acetonitrile increasing from 1% to 27%. The injection volume was 5 µL, containing approximately 2.5 µg of peptides per injection. The mass spectrometer was operated in data dependent mode; a high resolution (70,000 FWHM) MS scan (300 – 1,600 m/z) was performed to select the 12 most intense ions and fragmented using high energy C-trap dissociation for MS/MS analysis.

***Protein Search***

Raw data from LC-MS/MS was processed using the MaxQuant proteomics software package (version 2.0.3.0) ^29,30^ incorporating the Andromeda search engine ^31^. To identify peptides and proteins MS/MS spectra were matched against Uniprot Homo sapiens database (2021_03) containing 78,120 entries. All searches were performed using the default setting of MaxQuant, with trypsin as specified enzyme allowing two missed cleavages and a false discovery rate of 1% on the peptide and protein level. The database searches were performed with carbamidomethyl (C) as a fixed modification and acetylation (protein N terminus) and oxidation (M) as variable modifications. For the generation of label free quantitative (LFQ) ion intensities for protein profiles, signals of corresponding peptides different nano-HPLC MS/MS runs were matched by MaxQuant in a maximum time window of 1 minute ^32^.

***mtDNA amount quantification***

For DNA extraction, we used 20-30 mg of frozen skeletal muscle specimen. DNA was extracted using the AllPrep RNA, DNA, miRNA Universal Kit (QIAGEN, Nordic, Solletuna, Sweden. Mitochondrial DNA (MtDNA) amount was determined by quantitative real-time PCR (qPCR) of the mitochondrial cytochrome B (*MT-CYB*) and mitochondrial NADH dehydrogenase 5 (*MT-ND5*) gene normalized to the nuclear hemoglobin subunit beta (*HBB*) and beta 2-microglobulin (*B2M*) genes. The qPCR was performed using SYBR Green MasterMix (Thermo Fisher Scientific), 10 μM primers, and 10 ng of DNA template in a 384-well plate format using CFX384 Touch Real-TimePCR Detection Systems (Bio-Rad Laboratories). Thermal cycling included initial denaturation of 3 min at 95°C, 39 cycles of 10 s at 95°C and 30 s at 62°C, final extension of 10 s at 95°C, and melting curve analysis from 65° to 95°C with 0.5°C increments. Data were analyzed by qBASEplus version 3.0 (Biogazelle). The primer sequences were *MT-CYB* (Forward: 5’-GCCTGCCTGATCCTCCAAAT-3’, Reverse: 5’- AAGGTAGCGGATGATTCAGCC-3’), *MT-ND5* (Forward: 5’- AGGCGCTATCACCACTCTGTTCG-3’; Reverse: 5’- AACCTGTGAGGAAAGGTATTCCT-3’), *HBB* (Forward: 5’- CAGGTACGGCTGTCATCAGTTAG-3’; Reverse: 5’- CATGGTGTCTGTTTGAGGTTGCT-3’), *B2M* (Forward: 5’- GAGGCTATCCAGCGTACTCCA-3’; Reverse: 5’- CGGCAGGCATACTCATCTTTT-3’).

***Transmission Electron Microscopy (TEM)***

Samples for TEM analyses were fixed in 2.5% glutaraldehyde. For plastic embedding, they were then treated with 1% osmium tetroxide dehydrated in ethanol and embedded in epoxy resin. One-millimeter section was stained with methylene blue (0.5%, w/v) and boric acid (1%, w/v) and examined with a light microscope to mark the interesting areas for TEM analyses. Thereafter, 60- to 90-nm sections were cut on grids and stained with uranyl acetate and lead citrate by the Viikki Electron Microscopy Unit of the Institute of Biotechnology (EMBI, Biocenter Finland) and viewed with a JEM-1400 transmission electron microscope (Jeol).

From a subset (surgery cohort, n=17; lifestyle cohort, n=8), 15 TEM images were taken at each timepoint from the intermyofibrillar area. Participants were selected based on weight loss outcomes: individuals without (n=9) and with (n=8) type 2 diabetes with average weight loss were chosen from the surgery cohort, while those with the greatest weight loss were selected from the lifestyle cohort (n=8) in an effort to match the degree of weight loss observed in the surgery cohort. Each single mitochondrion was painted in Microscopy Image Browser ^33^, and morphological characteristics were extracted. The aspect ratio (ratio between the major and minor axes of the ellipse equivalent to the object) and the form factor [perimeter^2^/(4π × area)] were calculated representing mitochondrial elongation and interconnectivity. Roundness is the inverse of the aspect ratio (major axis/minor axis length) and is calculated as: 4 × area/(π × major axis^2^). Moreover, lipid droplets were painted and counted.
