# Supplementary material for: Human Skeletal Muscle Mitochondria Responses to Weight Loss Induced by Bariatric Surgery or Lifestyle Intervention
Source: Acta Physiol (Oxf). 2026 Jan 8;242(2):e70150. doi: 10.1111/apha.70150 (PMC12783452; doi:10.1111/apha.70150)
Supplement: Supplementary file 2 — Table S1: apha70150‐sup‐0002‐TableS1.docx. [file APHA-242-e70150-s008.docx]

| Supplementary table 1. Participant characteristics of the surgery cohort stratified for type 2 diabetes status at baseline | | | | | | | | |  |
| --- | --- | --- | --- | --- | --- | --- | --- | --- | --- |
|  | **Bariatric surgery – no T2D (n = 21)** | | | | **Bariatric surgery cohort + T2D (n = 18)** | | | |  |
|  | **baseline** | **5 months** | **12 months** | **P-value** | **baseline** | **6 months** | **12 months** | **P-value** | **p-value time*T2D** |
| Sex (females %) | 62% |  |  |  | 72% |  |  |  |  |
| Age (years) | 48.5 ± 7.8 |  |  |  | 44.9 ± 6.5 |  |  |  |  |
| Body weight (kg) | 130.5 ± 18.7 | 106.4 ± 15.0 | 102.1 ± 16.0 | **<0.001** | 131.9 ± 20.8 | 100.6 ± 16.2 | 95.5 ± 16.5 | **<0.001** | 0.242 |
| Waist (cm) | 131.0 ± 15.7 | 111.9 ± 9.6 | 107.7 ± 10.0 | **<0.001** | 136.6 ± 10.1 | 112.7 ± 11.2 | 107.1 ± 14.0 | **<0.001** | 0.476 |
| BMI (kg‎/m^2^) | 44.3 ± 5.2 | 36.2 ± 4.5 | 34.7 ± 4.8 | **<0.001** | 46.5 ± 6.1 | 35.5 ± 5.5 | 33.7 ± 5.7 | **<0.001** | 0.328 |
| Body fat (%) | 51.2 [45.4 :54.5] | 43.7 [38.0 :48.2] | 40.8 [34.6 :46.9] | **<0.001** | 49.4 [45.9 : 51.9] | 40.5 [35.1 : 45.8] | 36.3 [30.7 : 42.7] | **<0.001** | 0.678 |
| Body fat (kg) | 64.0 ± 14.2 | 45.4 ± 11.9 | 39.6 ± 12.3 | **<0.001** | 63.9 ± 12.1 | 39.4 ± 9.5 | 34.7 ± 11.2 | **<0.001** | 0.505 |
| Lean soft tissue mass (kg) | 58.7 [55.0 :67.0] | 56.0 [51.8 :63.1] | 55.2 [51.2 : 62] | 0.150 | 62.3 [56.2 : 68.9] | 55.0 [50.2 : 63.0] | 53.3 [50.2 : 62.6] | 0.182 | 0.869 |
| Appendicular lean soft tissue mass (kg) | 28.5 ± 5.2 | 26.0 ± 3.8 | 25.4 ± 4.0 | 0.065 | 28.5 ± 5.9 | 25.7 ± 5.2 | 25.0 ± 6.0 | 0.173 | 0.986 |
| Appendicular lean mass index | 9.6 ± 1.1 | 8.9 ± 0.7 | 8.6 ± 0.9 | **0.004** | 10.0 ± 1.4 | 9.0 ± 1.2 | 8.7 ± 1.3 | **0.018** | 0.859 |
| T2D present (number) | 0 | 0 | 0 |  | 18 | 9 | 5 |  |  |
| Fasting glucose (mmol/L) | 5.6 [5.5 : 6.3] | 5.4 [5.1 : 5.7] | 5.3 [5.1 : 5.4] | **0.002** | 6.8 [6.1 : 7.6] | 5.6 [5.1 : 5.9] | 5.7 [5.1 : 5.9] | **0.011** | 0.219 |
| Fasting insulin (mU/L) | 16.6 [13.5 :17.6] | 8.4 [4.5 : 12.1] | 5.7 [4.9 : 9.0] | **<0.001** | 17.5 [7.9 : 30.6] | 10 [6.1 : 11.7] | 9.0 [3.0 : 10.0] | **0.014** | 0.901 |
| HOMA-IR index | 4.2 [3.4 : 4.9] | 2.0 [1.1 : 3.2] | 1.4 [1.2 : 2.1] | **<0.001** | 6.1 [2.2 : 9.4] | 2.5 [1.5 : 3.0] | 2.2 [0.8 : 2.7] | **0.014** | 0.781 |
| Matsuda index | 2.3 [1.7 : 3.1] | 4.1 [2.7 : 5.0] | 5.3 [4.5 : 8.3] | **0.001** | 1.8 [1.0 : 3.8] | 3.4 [2.7 : 6.9] | 4.5 [2.8 : 8.2] | **0.034** | 0.603 |
| Total cholesterol (mmol/L) | 4.4 ± 0.8 | 3.9 ± 0.6 | 4.2 ± 1.1 | 0.124 | 4.5 ± 1.1 | 3.7 ± 0.8 | 3.8 ± 0.9 | **0.020** | 0.461 |
| HDL (mmol/L) | 1.3 ± 0.2 | 1.3 ± 0.2 | 1.5 ± 0.2 | **0.006** | 1.2 ± 0.26 | 1.1 ± 0.2 | 1.3 ± 0.3 | 0.247 | 0.288 |
| LDL (mmol/L) | 2.9 ± 0.7 | 2.2 ± 0.6 | 2.3 ± 0.8 | **0.014** | 2.8 ± 0.9 | 2.3 ± 0.7 | 2.2 ± 0.8 | 0.091 | 0.951 |
| TAG (mmol/L) | 1.1 [1.0 : 1.6] | 0.9 [0.8 : 1.2] | 0.8 [0.7 : 1.1] | **0.018** | 1.6 [1.1 : 2.1] | 1.3 [0.8 : 1.4] | 1.0 [0.9 : 1.2] | **0.004** | 0.698 |
| Work index | 2.5 ± 0.6 | 2.4 ± 0.5 | 2.5 ± 0.5 | 0.800 | 2.5 ± 0.6 | 2.6 ± 0.6 | 2.7 ± 0.6 | 0.783 | 0.663 |
| Sports index | 1.9 ± 0.7 | 2.3 ± 1.1 | 2.5 ± 0.9 | 0.078 | 2.2 ± 0.7 | 2.6 ± 0.7 | 2.4 ± 0.8 | 0.380 | 0.495 |
| Leisure index | 2.6 ± 0.6 | 3.0 ± 0.6 | 3.0 ± 0.7 | 0.110 | 2.8 ± 0.6 | 2.8 ± 0.5 | 3.0 ± 0.4 | 0.536 | 0.561 |
| Total physical activity (Baecke) | 7.2 ± 1.3 | 8.1 ± 1.5 | 8.3 ± 1.5 | 0.071 | 7.6 ± 1.3 | 8.1 ± 1.2 | 8.2 ± 1.1 | 0.467 | 0.648 |
| CRP (mg/L) | 4.9 ± 4.2 | 2.1 ± 2.0 | 1.2 ± 1.2 | **<0.001** | 7.0 ± 6.4 | 2.4 ± 3.2 | 1.2 ± 1.1 | **<0.001** | 0.331 |
| Leukocytes | 6.2 ± 1.5 | 5.7 ± 1.3 | 5.2 ± 1.2 | 0.069 | 7.2 ± 1.1 | 5.9 ± 1.2 | 5.8 ± 1.6 | **0.004** | 0.423 |
| Data are reported as mean ± SD (normally distributed variables) or median (interquartile range for skewed variables). P values were obtained using a generalized linear model using the REML method to study the effects of time on anthropometric and clinical parameters before and after weight loss intervention. We considered P < 0.05 significant. Skewed variables were log_e_-transformed before analysis. BMI, body mass index; HDL, high-density lipoprotein; LDL, low-density lipoprotein; TAG: triacylglycerol; HOMA-IR, homeostatic model for the assessment of insulin resistance; CRP, C-reactive protein. | | | | | | | | | |
