# Supplementary material for: Human Skeletal Muscle Mitochondria Responses to Weight Loss Induced by Bariatric Surgery or Lifestyle Intervention
Source: Acta Physiol (Oxf). 2026 Jan 8;242(2):e70150. doi: 10.1111/apha.70150 (PMC12783452; doi:10.1111/apha.70150)
Supplement: Supplementary file 11 — Figures S1–S4: apha70150‐sup‐0012‐FiguresS1‐S4.zip. [file APHA-242-e70150-s009.zip › APHA70150_Figure captions.docx]

Figure S1: Top 5 biological pathways for mitochondria-related protein changes upon surgery-induced and lifestyle-induced weight loss interventions. Protein enrichment for biological pathways using differentially expressed mitochondria-related proteins selected from MitoMiner 4.0 (nominal *p* < 0.05) associated with weight loss percentage at the respective timepoint following (A) surgery-induced weight loss (*n* = 33) and (B) lifestyle-induced weight loss (*n* = 19). The top 5 significant findings from the ingenuity pathway analysis (IPA) tool (*p* < 0.05) are shown. For several pathways, IPA provided z-scores for pathway directionality by calculating the observed number of “activated” genes (z-score > 0), “inhibited” genes (z-score < 0), or no directionality prediction (z-score = 0). Z-scores > 2 or < −2 were considered statistically significant for directionality. Results ranked according to statistical significance.

Figure S2: Top 5 biological pathways for protein changes upon surgery-induced weight loss stratified for type 2 diabetes status. Protein enrichment for biological pathways using differentially expressed proteins associated with weight loss percentage at respective timepoint in (A) people without type 2 diabetes (*n* = 18) and (B) with type 2 diabetes (*n* = 15). The top 5 significant findings from the ingenuity pathway analysis (IPA) tool (*p* < 0.05) are shown. For several pathways, IPA provided z-scores for pathway directionality by calculating the observed number of “activated” genes (z-score > 0), “inhibited” genes (z-score < 0), or no directionality prediction (z-score = 0). Z-scores > 2 or < −2 were considered statistically significant for directionality. Results ranked according to statistical significance.

Figure S3: Skeletal muscle mitochondrial and lipid droplets number and morphology following surgery- and lifestyle-induced weight loss interventions. TEM images of muscle intermyofibrillar mitochondria (M) and lipid droplets (L) from three representative study participants before and after (A) lifestyle-induced weight loss and (B) and after surgery-induced weight loss without (C) and with type 2 diabetes. Magnification, ×2000. Scale bars, 2 μm. Quantification of (D) the number of mitochondria, (E) mitochondrial surface area per total muscle fiber area, (F) average eccentricity of mitochondria, (G) average perimeter of mitochondria, (H) aspect ratio (the length-to-width ratio of mitochondria), (I) form factor (the branching of mitochondria), (J) lipid droplet number and (K) average lipid droplet area in skeletal muscle. The bars indicate the mean ± SD and dots indicate individual measurements. Three bars on the left indicate lifestyle cohort (*n* = 8), three bars in the middle indicate people without type 2 diabetes in the surgery cohort (*n* = 9) and three bars on the right indicate people with type 2 diabetes (*n* = 8).

Figure S4: Skeletal muscle mtDNA amount and mitochondrial respiration stratified for type 2 diabetes. Relative mtDNA amount to nuclear DNA content measured by amplifying (A) CYTB and (B) ND5 mitochondrial gene areas with qPCR. The bars indicate the mean ± SD and dots indicate individual measurements. Left three bars: lifestyle cohort (CRYO, *n* = 19), middle three bars indicate people without type 2 diabetes and right three bars indicate people with diabetes (RYSA, *n* = 39). * *p* < 0.05 (generalized linear model with time point as explanatory variable and Bonferroni post hoc correction). (C-F) Mitochondrial respiration as stratified by type 2 diabetes status. Data are shown for individuals without diabetes (C,D, *n* = 4) and with diabetes (E,F *n* = 4). Added substrates and inhibitors for respiration measurements are shown in chronological order over the plot. PGM: pyruvate, glutamate, malate; S: succinate; Omy: oligomycin; FCCP: Carbonylcyanide-4-(trifluoromethoxy)-phenylhydrazone; Rot; rotenone.
