# Supplementary figures and images for: Human Skeletal Muscle Mitochondria Responses to Weight Loss Induced by Bariatric Surgery or Lifestyle Intervention
Source: Acta Physiol (Oxf). 2026 Jan 8;242(2):e70150. doi: 10.1111/apha.70150 (PMC12783452; doi:10.1111/apha.70150)

**A**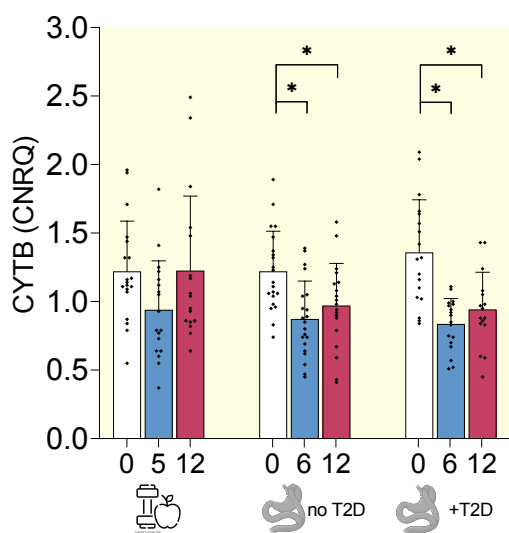**B**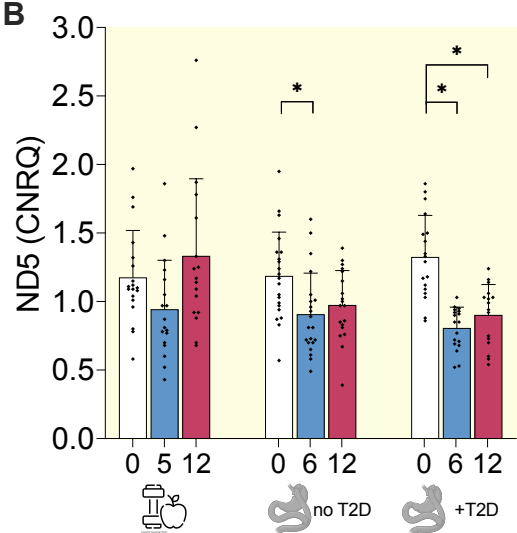**C**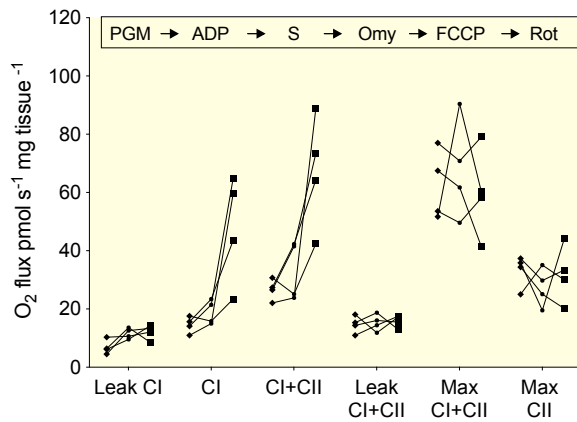**D**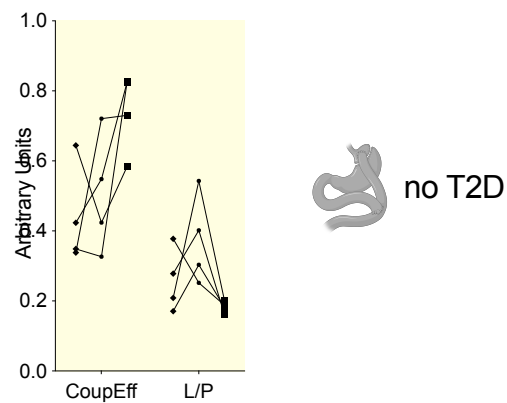**E**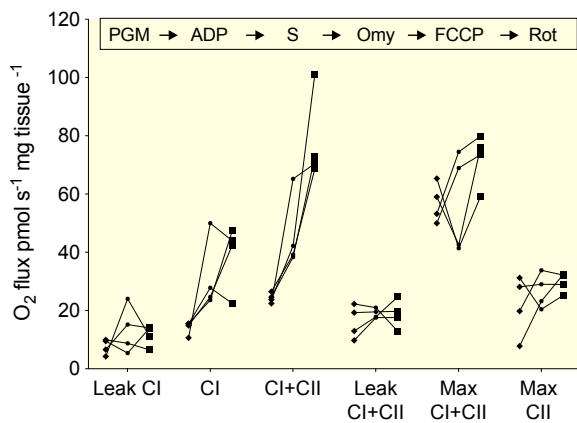**F**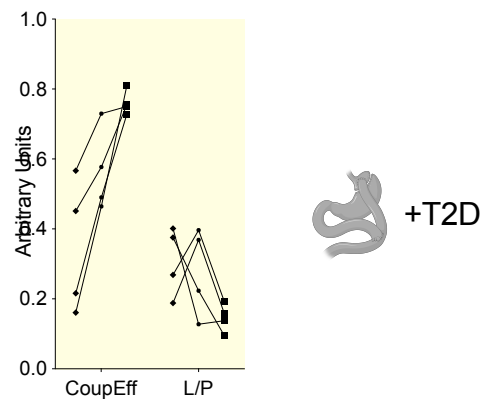

Supplement: Supplementary file 11 — Figures S1–S4: apha70150‐sup‐0012‐FiguresS1‐S4.zip. [file APHA-242-e70150-s009.zip › apha70150-sup-0014-FigureS4.pdf]

**A****6 months**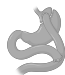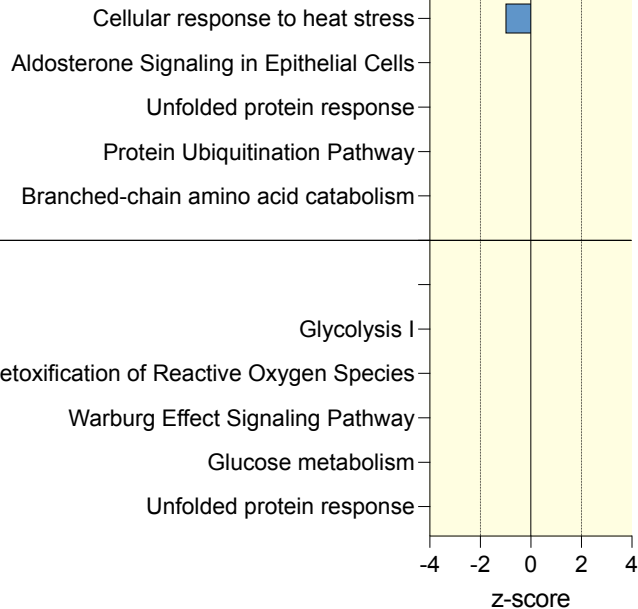**B****5 months**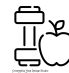**12 months**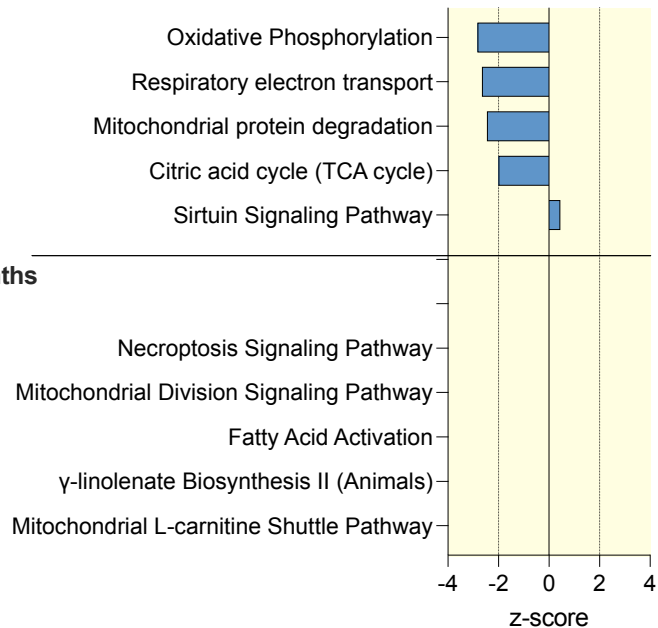

Supplement: Supplementary file 11 — Figures S1–S4: apha70150‐sup‐0012‐FiguresS1‐S4.zip. [file APHA-242-e70150-s009.zip › apha70150-sup-0011-FigureS1.pdf]

**A**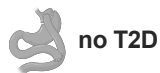**6 months**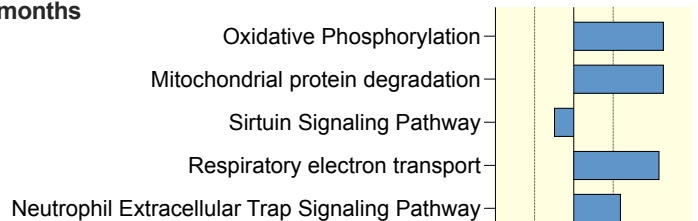**12 months**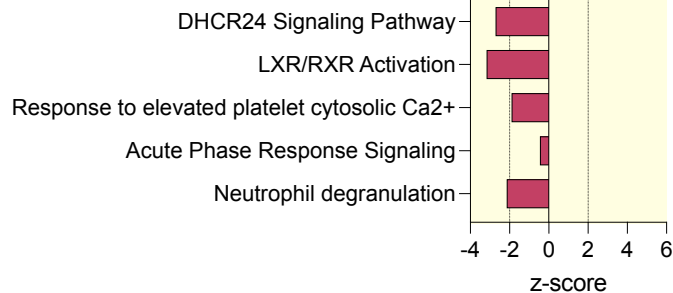**B**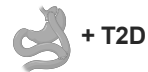**6 months**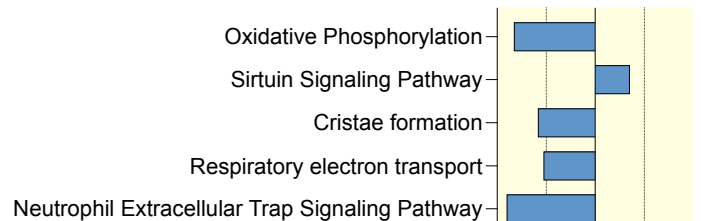**12 months**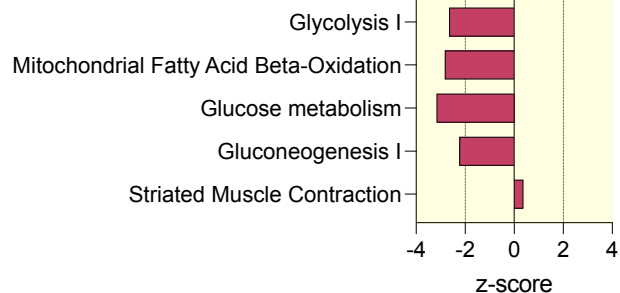

Supplement: Supplementary file 11 — Figures S1–S4: apha70150‐sup‐0012‐FiguresS1‐S4.zip. [file APHA-242-e70150-s009.zip › apha70150-sup-0012-FigureS2.pdf]

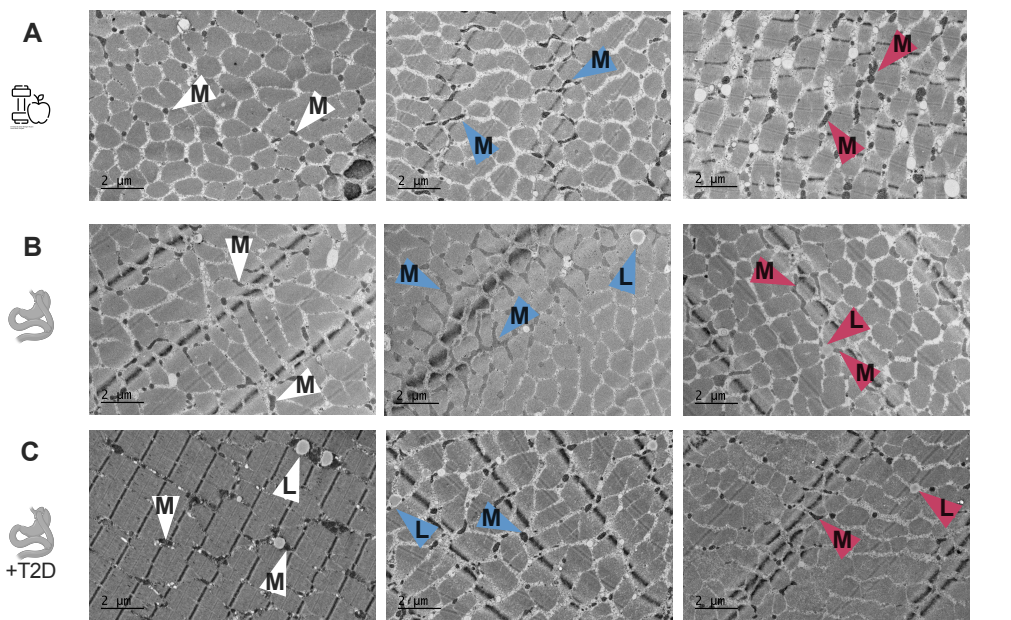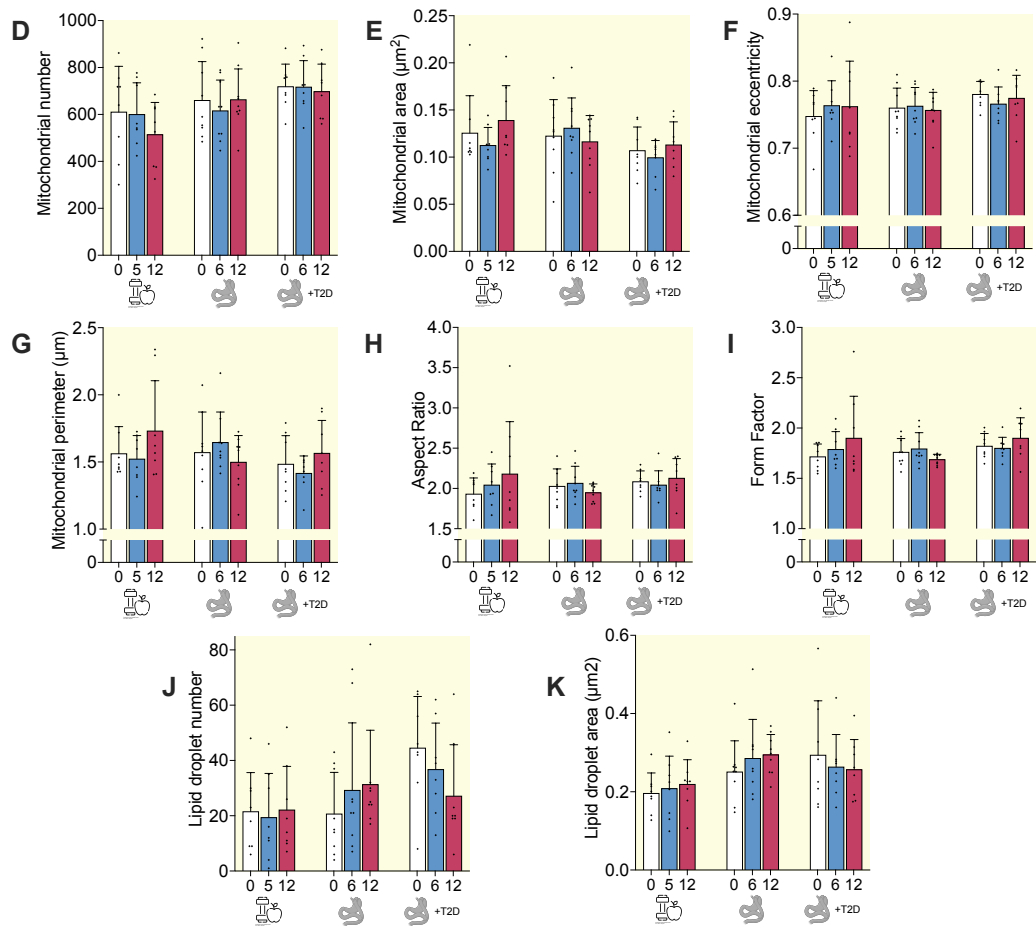

Supplement: Supplementary file 11 — Figures S1–S4: apha70150‐sup‐0012‐FiguresS1‐S4.zip. [file APHA-242-e70150-s009.zip › apha70150-sup-0013-FigureS3.pdf]
